# GacA regulates symbiosis and mediates lifestyle transitions in *Pseudomonas*

**DOI:** 10.1128/msphere.00277-25

**Published:** 2025-09-08

**Authors:** Youqing Luo, Apsara Srinivas, Casey Guidry, Carolee Bull, Cara H. Haney, Corri Hamilton

**Affiliations:** 1 Department of Microbiology and Immunology, University of British Columbia 8166, Vancouver, Canada; 2 Department of Biological Sciences, University of Pittsburgh 6614, Pittsburgh, Pennsylvania, USA; 3 Department of Plant Pathology and Environmental Microbiology, Pennsylvania State University, State College 8082, University Park, Pennsylvania, USA; Ben-Gurion University, Negev, Beer-Sheva, Israel

**Keywords:** gene regulation, two-component system, plant-microbe interactions, *Pseudomonas*, plant pathogenic bacteria

## Abstract

Through horizontal gene transfer, closely related bacterial strains assimilate distinct sets of genes, resulting in significantly varied lifestyles. However, it remains unclear how strains properly regulate horizontally transferred virulence genes. We hypothesized that strains may use components of the core genome to regulate diverse horizontally acquired genes. To investigate how closely related bacteria assimilate and activate horizontally acquired DNA, we used a model consisting of strains in the *brassicacearum*/*corrugata*/*mediterranea* (BCM) subclade *of Pseudomonas fluorescens*, including *Pseudomonas* species N2E2 and N2C3, which exhibit contrasting lifestyles on the model plant *Arabidopsis. Pseudomonas* sp. N2E2 is a plant commensal and contains genes encoding biosynthetic enzymes for the antifungal compound 2,4-diacetylphloroglucinol (DAPG). In contrast, *Pseudomonas* sp. N2C3 lacks DAPG biosynthesis and has gained a pathogenic island encoding syringomycin (SYR)- and syringopeptin (SYP)-like toxins from the plant pathogen *Pseudomonas syringae*. This causes a transition in lifestyle from plant-protective N2E2 to plant-pathogenic N2C3. We found that N2E2 and N2C3 share a highly conserved two-component system GacA/S, a known regulator of DAPG and SYR/SYP. Using knockout mutations, we found that a *ΔgacA* mutation resulted in loss of expression of SYR/SYP virulence genes and returned pathogenic N2C3 to a plant commensal lifestyle. Our study further explored the conservation of regulatory control across strains by demonstrating that GacA genes from both distant and closely related *Pseudomonas* strains could functionally complement one another across the genus.

**IMPORTANCE:**

Emerging pathogens represent a significant threat to humans, agriculture, and natural ecosystems. Bacterial horizontal gene transfer (HGT) aids in the acquisition of novel genes that facilitate adaptation to new environments. Our work shows a novel role for GacA in orchestrating the regulatory changes necessary for virulence and lifestyle transitions facilitated by HGT. These findings suggest that the GacA/S system plays a key role in mediating transitions across diverse *Pseudomonas* symbiotic lifestyles. This work provides insights into the mechanisms that drive the emergence of pathogenic strains and highlights potential targets for managing bacterial threats to plant health.

## INTRODUCTION

Horizontal gene transfer (HGT) is a powerful evolutionary mechanism that enables bacteria to acquire virulence traits and adapt to new hosts, facilitating ecological transitions and the emergence of novel pathogenic strains (1–3). Unlike vertical inheritance, HGT allows bacteria to integrate genetic material directly from other species or environmental sources, leading to dramatic shifts in bacterial behavior, including the acquisition of genes associated with antibiotic resistance, metabolic versatility, and virulence (2). Beyond simply enabling adaptation, HGT promotes genomic plasticity, allowing shifts between commensal and pathogen. This regulatory flexibility makes HGT a key driver of bacterial evolution and emerging pathogenic threats.

The *Pseudomonas fluorescens brassicacearum*/*corrugata*/*mediterranea* (BCM) subclade exemplifies how HGT contributes to host specialization and lifestyle shifts. Strains within the BCM subclade share 99% 16S rRNA identity and span a spectrum of lifestyles, from beneficial to pathogenic, in association with diverse hosts. The BCM subclade includes WCS365, NFM421, DF41, and N2E2, which are commensal strains that promote root growth and protect plants from diverse fungal and bacterial pathogen infections (4–8). However, it also includes pathogenic strains including *P. corrugata,* the causative agent of tomato pith necrosis (9) and *Pseudomonas* spp. N2C3 and R401 cause root stunting on gnotobiotic *Arabidopsis* (10, 11). Plant pathogenic strains in the BCM subclade use a lipopeptide toxin-based virulence strategy that results in disease on diverse hosts ranging from plant species from the families *Brassicaceae* and *Papaveroideae* (9, 10). Studies on *Pseudomonas* sp. N2C3 shows that this strain acquired the toxin-encoding pathogenicity islands while losing other non-pathogenic gene clusters through HGT (10).

One central challenge in understanding the rise of new pathogens is uncovering how bacteria regulate these newly acquired genes to ensure they function cohesively with existing cellular processes. While the mechanics of HGT integration are well understood, an open question remains: how are these foreign genes effectively regulated to ensure functionality within their new host? Bacteria rely on two-component system (TCS), like the GacS/GacA system, to sense and respond to environmental signals, modulating gene expression accordingly (12). In *Pseudomonas* spp., the GacS/GacA system regulates hundreds of genes, through the Rsm family of small trans-acting regulatory RNAs, including those tied to plant pathogenicity and biocontrol (12, 13). In beneficial strains like *P. protegens* CHAO and Pf-5, GacA regulates antifungal secondary metabolites such as 2,4-diacetylphloroglucinol (DAPG) (14, 15), while in pathogens like *Pseudomonas syringae* pv. *syringae*, GacA modulates virulence traits including the type III secretion system (T3SS) (16, 17). Many studies have investigated the role T3SSs play in the pathogenicity of both plant- and animal-associated bacteria, functioning as a specialized export mechanism for effector proteins that manipulate host responses (18). However, in the BCM subclade of *P. fluorescens*, non-pathogenic strains have genes that encode T3SSs, whereas pathogenic strains have acquired phytotoxins like syringomycin (SYR) and syringopeptin (SYP) (10), suggesting an evolutionary trade-off and reprogramming of bacterial lifestyle. This led us to hypothesize that GacA/S may regulate newly acquired virulence genes in both pathogenic and commensal strains facilitating lifestyle transitions between closely related strains.

This study examines how closely related strains within the BCM subclade of *P. fluorescens* utilize a functional GacA/S system to regulate horizontally acquired genes and facilitate transitions between commensal and pathogenic lifestyles. By examining the gain and loss of *gacA*, we investigated how these genetic changes drive transitions between pathogenesis and commensalism. We used genetic complementation across distantly related *Pseudomonas* strains and characterized spontaneous mutations to assess GacA’s role in these lifestyle shifts. By focusing on a subclade with both commensals and pathogens, we reveal how GacA regulates newly acquired genes to shape ecological roles and govern the evolution of bacterial phenotypic changes. Through advancing our understanding of these regulatory mechanisms, we aim to inform strategies for managing emerging pathogens and protecting plant health.

## RESULTS

### GacA regulates horizontally acquired pathogenicity and specialized metabolite biosynthesis genes in *Pseudomonas*

**Fig 1:**
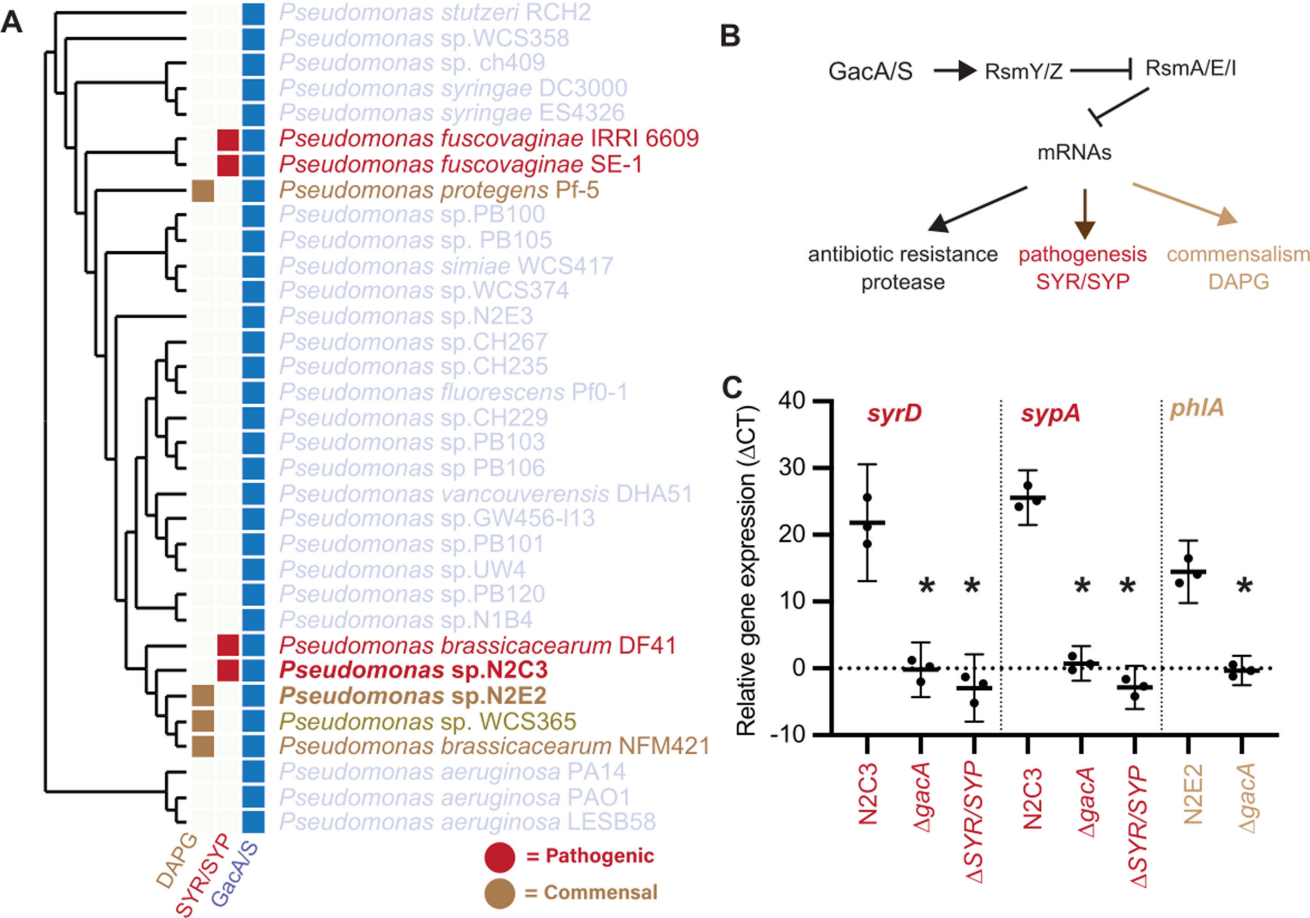
The presence of a functional GacA/S system is necessary for expression of horizontally transferred genomic islands in *Pseudomonas* spp. (**A**) A phylogenetic tree based on 122 core *Pseudomonas* genes shows conservation of GacA/S, while DAPG and SYR/SYP are gained and lost throughout the genus. (**B**) Model of the hypothesis of the role of Gac-Rsm in the regulation of horizontally acquired genomic elements. (**C**) ΔCT values of genes *syrD* (syringomycin), *sypA* (syringopeptin), *phlA* (2,4-diacetylphloroglucinol), and compared to the *recA* housekeeping gene in Kings B Media. Each dot represents an average of three technical replicates and one biological replicate (*n*  =  9). Shown are mean ± standard deviation, and an asterisk for significance (*P*  <  0.05) in a Student’s *t*-test.

To better understand how otherwise commensal strains can effectively utilize pathogenic genomic elements gained through HGT, we characterized strains in the BCM subclade of *P. fluorescens* with distinct plant-associated lifestyles. We focused on two closely related isolates from groundwater: a plant pathogen *Pseudomonas* sp. N2C3 and a plant commensal *Pseudomonas* sp. N2E2 (10, 19). Although N2C3 and N2E2 are part of distinct, currently unnamed species in the BCM subclade (20), these strains have sufficiently similar 16S rRNA sequences that amplicon sequencing would assign them to the same operational taxonomic unit (10). We found that while *Pseudomonas* GacA/S predicted amino acid sequences are highly conserved within the BCM subclade (>99% identical for GacA and >98% for GacS) and across the genus *Pseudomonas* (>88% identical for GacA and >70% identical for GacS), downstream mobile elements including the antimicrobial DAPG and the SYR/SYP toxins show gain and loss even between the closely related strains *Pseudomonas* spp. N2E2 and N2C3 (10) (Fig. 1A; Fig. S1 and S2). Both N2E2 and N2C3 use these antimicrobial compounds to facilitate ecological stability within a host-associated niche, making them important contributors to symbiotic capacity and host health.

To determine whether *gacA/S* regulates horizontally acquired islands in the BCM subclade*,* including SYR/SYP (Fig. 1B), we generated deletions of *gacA* in N2E2 and N2C3 and tested whether the loss of *gacA* affects the expression of genes encoding biosynthetic enzymes for DAPG, SYR/SYP, and the associated antifungal and plant virulence phenotypes. Using qRT-PCR, we found that the deletion of *gacA* in *Pseudomonas* sp. N2C3 results in decreased expression of *syrD* and *sypA,* required for biosynthesis of SYR and SYP, respectively (Fig. 1C). The deletion of *gacA* in N2E2 resulted in decreased *phlA* required for biosynthesis of DAPG (Fig. 1C). These results confirm that *gacA* expression is required for expression of horizontally transferred symbiosis factors that regulate both beneficial and pathogenic traits with the BCM subclade of *P. fluorescens*.

**Fig 2:**
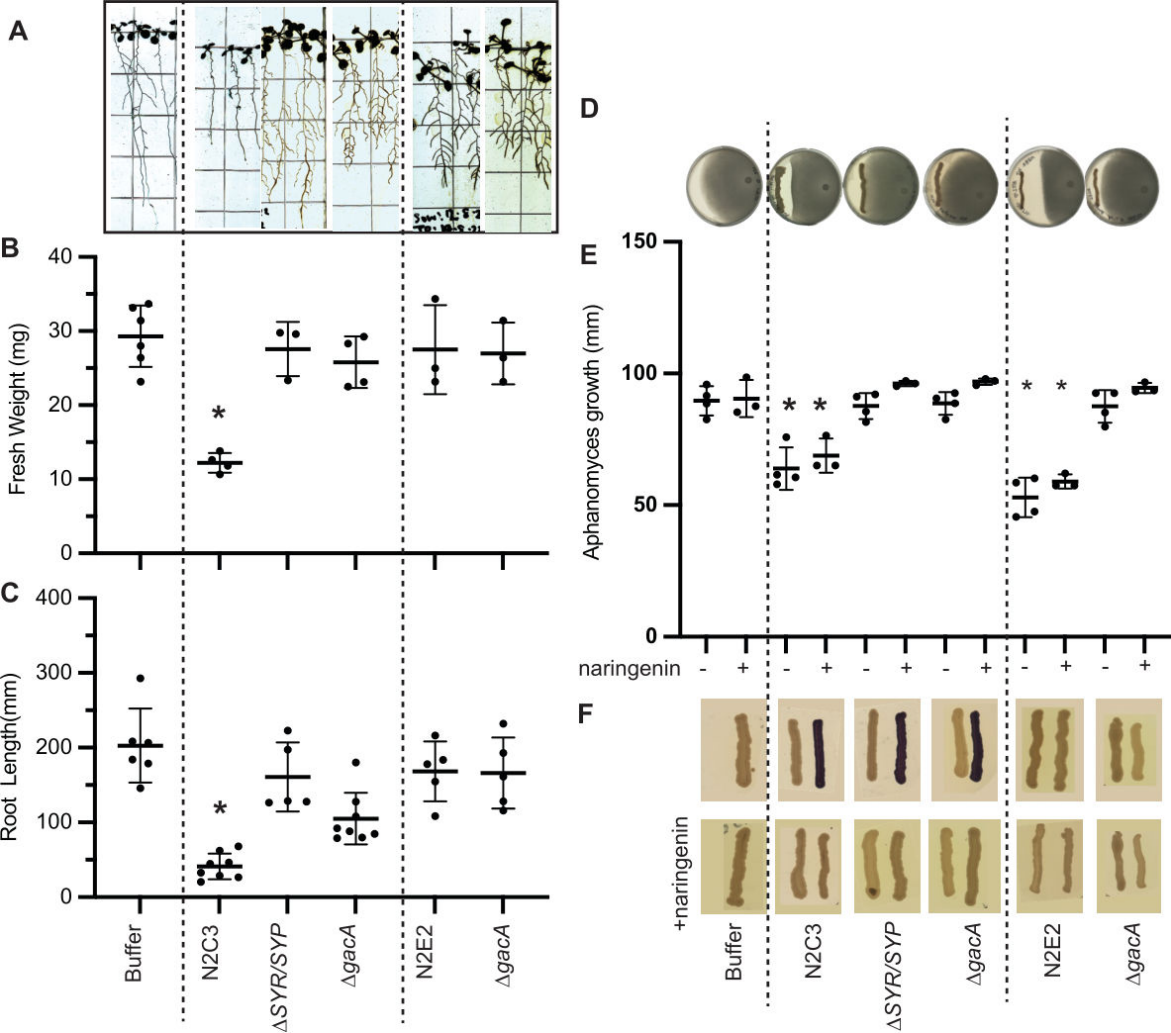
GacA controls lifestyle-dependent phenotypes independent of quorum signaling. (**A**) Representative images of gnotobiotic *Arabidopsis* seedlings inoculated with Buffer or *Pseudomonas* spp. N2C3, N2C3 *∆gacA*, N2C3 *∆syr/syp,* N2E2, or N2E2 *∆gacA*. (**B**) Plant fresh weight and (**C**) seedling root length were quantified. Each dot represents the average of three to five plants, and three to six biological replicates were performed for each treatment (*n*  =  9–30). Mean ± standard deviation is shown, and an asterisk indicates significant (*P*  <  0.05) differences as determined by a one-way ANOVA followed by a post hoc Tukey HSD test. (**D, E**) *A. euteiches* AE-1 antagonism assay competed against *Pseudomonas* spp. N2C3, N2C3 *∆gacA*, N2C3 *∆syr/syp*, N2E2, N2E2 *∆gacA,* or Buffer. (D) Representative images of *A. euteiches* growth (right side of plates) with the *Pseudomonas* strain (left) without naringenin. (**E**) Quantification of *A. euteiches* growth when challenged with *Pseudomonas* spp. strains in the presence (+) or absence (−) of naringenin. Experiments were repeated four independent times, with three plates per replicate (*n*  =  9–12). (**F**) Representative images showing qualitative quorum sensing activity, reported through the purple violacein pigment produced by *Chromobacterium violaceum* CV026 AHL biosensor, presented second in each panel.

We then evaluated whether a loss of *gacA* would also affect the virulence and antimicrobial activities of N2C3 and N2E2. Consistent with its role in regulating the expression of SYR/SYP*,* we found that an N2C3 *∆gacA* mutant loses virulence as measured by root stunting and reduced plant fresh weight (Fig. 2A through C). Consistent with the absence of SYR/SYP and other plant virulence factors in the commensal strain N2E2, we found no difference in root length and fresh weight in an N2E2 ∆*gacA* mutant (Fig. 2A through C). Similarly, we found that while wild-type N2C3 and N2E2 inhibit the growth of the oomycete root-rot pathogen *Aphanomyces euteiches* AE-1, the *∆gacA* mutants in both N2E2 and N2C3 lose the ability to inhibit AE-1 growth (Fig. 2D and E). Collectively, these findings suggest that GacA may facilitate the regulation of genes within horizontally transferred islands, including acquired SYR/SYP and DAPG genes, which have been evolutionarily gained and lost in *Pseudomonas* spp (10).

SYR/SYP biosynthesis is regulated by a C6-acyl homoserine lactone (AHL) perceived by an associated LuxR regulator that is co-inherited with the SYR/SYP island (10). Some GacA/S-dependent processes are quorum dependent (21), suggesting that GacA/S may indirectly regulate SYR/SYP biosynthesis by regulating the associated *luxR/I* genes. We assessed whether GacA regulates SYR/SYP and DAPG through quorum sensing by asking whether the N2C3 ∆*gacA* mutant still produces C6-AHL. We used *Chromobacterium violaceum* CV026 C6-AHL reporter that turns purple in the presence of C6-AHLs; N2C3, but not N2E2, was previously shown to activate this reporter (10). We found that the N2C3 *∆gacA* mutant could still induce the reporter, indicating that it still makes C6-AHL (Fig. 2F). N2E2, in contrast, lacks production of C6-AHL and did not activate the reporter (Fig. 2F). To further investigate whether quorum sensing affects the production of SYR/SYP, DAPG, and consequentially inhibition of oomycete pathogen growth, we used the quorum quenching flavanone naringenin, which reduces AHL levels (22). While naringenin suppressed N2C3-mediated activation of the CV026 reporter *in vitro* (Fig. 2F), we found that both N2C3 and N2E2 were able to inhibit *AE-1* growth in the presence of naringenin (Fig. 2E). These data indicate that the production of DAPG and SYR/SYP secondary metabolites, and GacA regulation of SYR/SYP and DAPG, are independent of quorum signaling mechanisms (Fig. 2E and F).

### GacA functions in shared symbiotic traits independent of bacterial lifestyle

**Fig 3:**
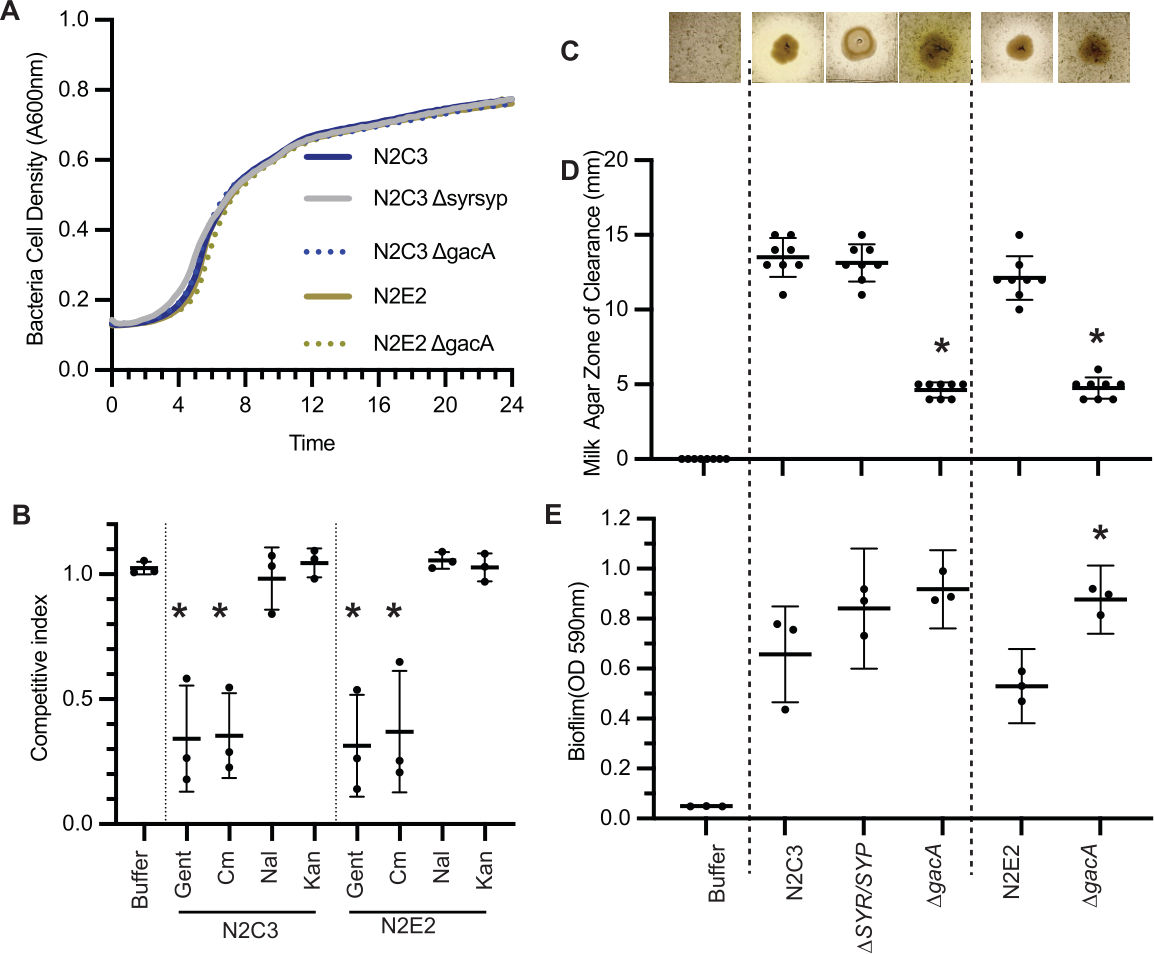
GacA controls the regulation of conserved symbiosis factors in closely related strains with divergent lifestyles. (**A**) Twenty-four hours growth curves of each strain were performed in LB media with no significant differences in growth over time via repeated measures of ANOVA (*P*  <  0.05; *n*  =  9). (**B**) To test whether *gacA* is differentially required for antibiotic resistance, we grew N2C3, N2C3 *∆gacA*, N2E2, or N2E2 *∆gacA* in subinhibitory concentrations of multiple antibiotics and described growth outcome as the competitive index of *∆gacA* strains compared to the wild-type parent strain. (**C, D**) To test whether *gacA* is necessary for protease activity, 5 µL of 0.02 OD_600nm_ bacterial cultures were spotted on milk agar plates. (**C**) Representative images of N2C3 and controls on milk agar. (**D**) Quantification of the zone of clearance for *Pseudomonas* spp. N2C3, N2C3 *∆syr/syp*, N2C3 *∆gacA*, N2E2, or N2E2 *∆gacA*. Each dot represents an average of three spots. (**E**) To test whether *gacA* is necessary for biofilm formation, a PVC-crystal violet assay was performed and biofilm formation was quantified colorimetrically via absorbance 590. (**B, D, E**) Experiments were repeated three to six independent times (*n*  =  9–20). Mean shown and asterisk indicate significant (*P*  <  0.05) differences as determined by a one-way ANOVA, followed by a post hoc Tukey HSD test.

Because we found that GacA regulates lifestyle-dependent horizontally transferred islands, we hypothesized that it also regulates a shared set of genes that influence symbiosis independent of lifestyle. Importantly, we found that mutations in *gacA* do not lead to overall growth defects in minimal media and rich media as indicated by similar doubling times and area under the curves for wild-type and *gacA* mutants (Fig. 3A and Fig. S3). GacA is known to regulate antibiotic resistance in pathogenic *Pseudomonas* strains (23); antibiotic resistance and microbe-microbe competition promote survivability in the context of high-density host-associated microbial communities (24). We found that in *Pseudomonas* sp. N2C3 and N2E2, loss of *gacA* increases sensitivity to subinhibitory concentrations of gentamycin and chloramphenicol (Fig. 3B and Table S1). Protease activity and biofilm formation are important for symbiosis; biofilm formation is required for plant colonization and virulence (25), while protease activity is required for rhizosphere competition and utilized to lyse a portion of the cell walls of pathogenic fungi (26). To quantify protease activity, we used a milk agar assay and found that GacA is necessary for protease activity in both *Pseudomonas* spp. N2C3 and N2E2 (Fig. 3C and D). Using a crystal violet assay, we found that the loss of *gacA* significantly increases biofilm formation in N2E2 and trends toward being increased in N2C3 (*P* = 0.093) (Fig. 3E). These data suggest that GacA plays a role in shared functions that shape symbiosis but is not required to maintain overall growth.

### Complementation of GacA between strains restores symbiotic functions

**Fig 4:**
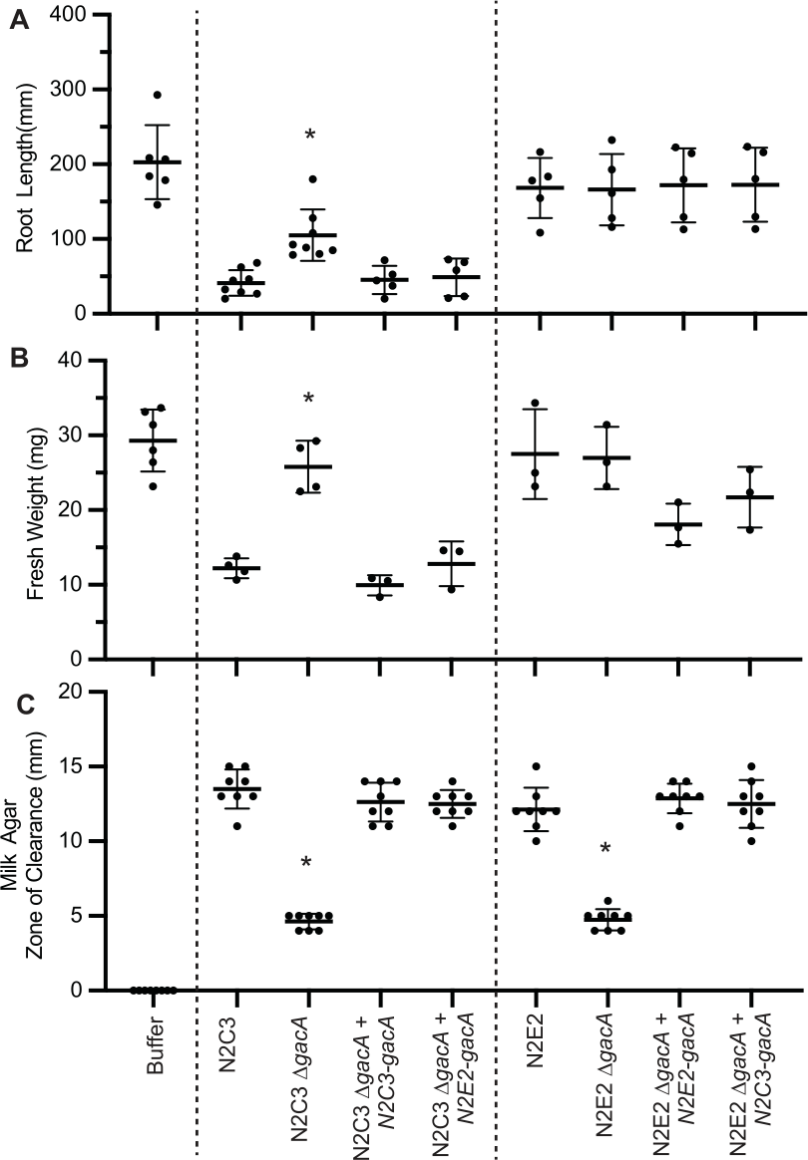
GacA can heterologously complement *Pseudomonas* with distinct lifestyles. To test whether virulence phenotypes in N2C3 can be complemented by *gacA* from N2E2, gnotobiotic *Arabidopsis* seedlings were inoculated with *Pseudomonas* spp. N2C3, N2C3 *∆gacA*, express *gacA* under its native promoter *N2C3-gacA_pro_:N2C3-gacA* or *N2E2-gacA_pro_:N2E2-gacA*. Differences were quantified using (**A**) seedling root length and (**B**) plant fresh weight. Each dot represents the average of three to five plants over three to six biological replicates (*n*  =  9–30). Mean ± standard deviation is shown, and asterisks indicate significant (*P*  <  0.05) differences as determined by a one-way ANOVA, followed by a post hoc Tukey HSD test. (**C**) To test whether *gacA* is sufficient for protease activity, 5 µL of 0.02 OD_600nm_ cultures was spotted on milk agar plates, and the zone of clearance was measured. Mean ± standard deviation is shown and asterisks indicate significant (*P*  <  0.05) differences as determined by a one-way ANOVA, followed by a post hoc Tukey HSD test.

To determine if a single GacA protein can regulate genetic islands from both pathogenic and beneficial strains, we asked whether *gacA* from the N2C3 pathogenic strain could complement an N2E2 *∆gacA* commensal mutant and vice versa. We generated constructs expressing *gacA* under its native promoter on a plasmid. The N2C3 and N2E2 predicted GacA amino acid sequences are 100% identical (Fig. S1), and the *gacA* DNA sequences, including the promoters, retain 99% identity; thus, we expected the *gacA* genes to functionally complement. However, it is possible that the slight differences are important for the regulation of diverse horizontally transferred islands. As expected, *N2C3-gacA_pro_:N2C3-gacA* complemented the *in planta* N2C3 *∆gacA* phenotype to wild-type-like levels for root length, fresh weight, and protease activity (Fig. 4A through C). Likewise, *N2E2-gacA_pro_:N2E2-gacA* complemented the protease activities of the N2E2 *∆gacA* mutant back to N2E2 wild-type levels (Fig. 4C). We found that the introduction of the beneficial *N2E2-gacA_pro_:N2E2-gacA* construct into the N2C3 *∆gacA* mutant also complemented protease activity to the same level as *N2C3-gacA_pro_:N2C3-gacA* construct (Fig. 4A through C). This held true for the introduction of *N2C3-gacA_pro_:N2C3-gacA* into the N2E2 *∆gacA* mutant, which restored the protease activity to N2E2 wild-type levels (Fig. 4A through C). The ability of GacA to regulate lifestyle-specific traits in diverse strains underscores its importance in mediating transitions between different lifestyles.

### GacA can heterologously regulate the expression of virulence factors across *Pseudomonas* species

**Fig 5:**
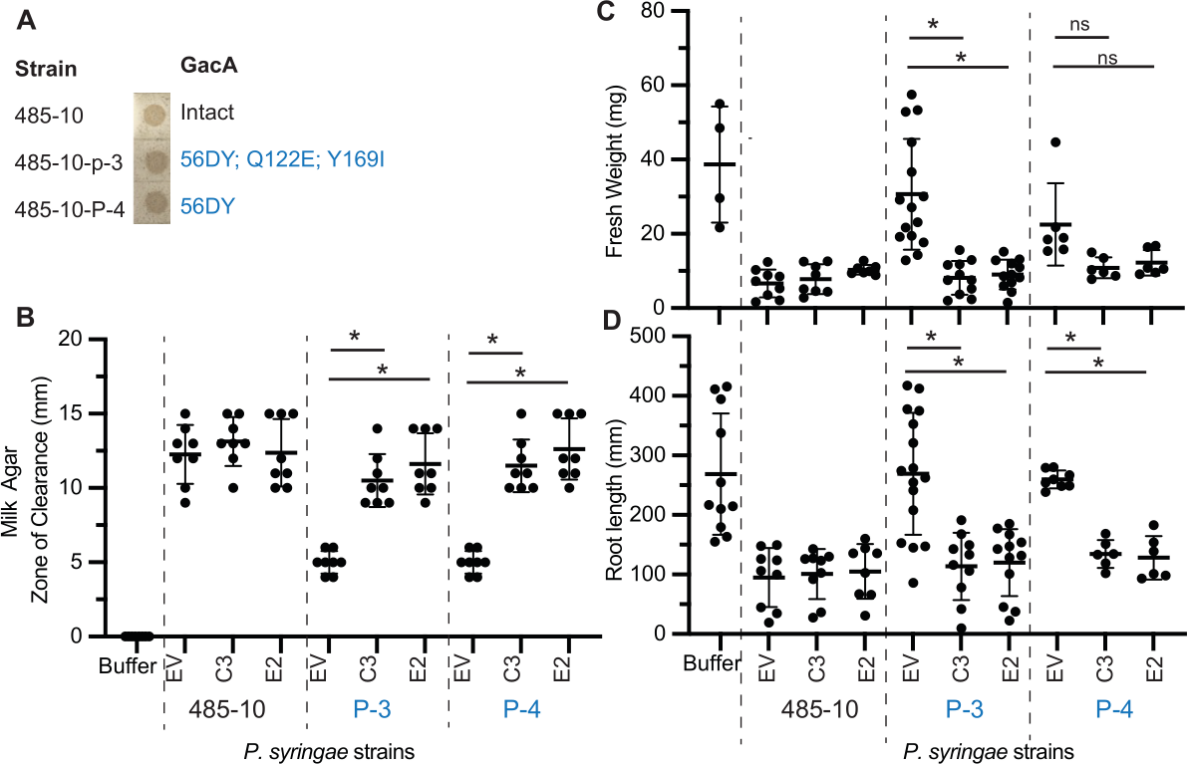
*GacA* from *P. fluorescens* BCM subclade strains can heterologously complement *P. syringae* 485-10 *gacA* mutants. (**A**) Two spontaneous protease-negative mutants in *P. syringae* 485-10 were identified. The *gacA* genotype is indicated by Sanger sequencing. (**B**) Protease activity, (**C**) plant fresh weight, and (**D**) root growth inhibition of 485-10 with introduction of empty vector (EV), *N2C3-gacA_pro_:N2C3-gacA* (C3) or *N2E2-gacA_pro_:N2E2-gacA* (E2). Mean ± standard deviation is shown, and asterisks indicate significant (*P*  <  0.05) differences as determined by a one-way ANOVA, followed by a post hoc Tukey HSD test.

To determine if GacA from N2C3 or N2E2 can regulate virulence in distantly related *Pseudomonas* spp., we tested whether *gacA* from BCM strains could heterologously regulate gene expression in a syringomycin-producing *P. syringae* strain 485-10, originally isolated from citrus blast infected tissues (27). The *Pseudomonas* spp. N2E2 and N2C3 GacA predicted amino acid sequence is 92.8% identical to that of *P. syringae* DC3000 (Fig. S1); thus, *P. fluorescens* BCM subclade and *P. syringae* have some of the most divergent *gacA* sequences in the genus. We found that *P. syringae* 485-10 showed protease activity on milk agar, indicating it has a functional GacA/S signaling pathway (Fig. 5A). We found that, like syringomycin-producing N2C3 and *P. fuscovaginae* SE-1, *P. syringae* 485-10 caused reduced fresh weight when inoculated onto *Arabidopsis* seedlings (Fig. 5B).

To identify GacA mutants, two spontaneous protease activity-negative *P. syringae* 485-10 mutants were identified by screening on milk agar (Table S2). Spontaneous protease-negative mutants 485-10-P-3 and 485-10-P-4 lost the ability to clear milk agar, resulting in a reduced zone of clearance compared to the respective parent strains (Fig. 5A and C). By PCR amplification of the *gacA* gene and Sanger sequencing, we found that spontaneous protease mutants 485-10-P-3 and 485-10-P-4 had missense mutations in *gacA* (Fig. 5A). Consistent with a loss of GacA/S signaling, when inoculated onto plants, all protease-negative mutants resulted in higher fresh weight compared to their respective parent strains, indicating a loss of virulence (Fig. 5B).

To determine if a BCM *gacA* gene could regulate virulence traits in *P. syringae,* we tested whether N2C3 and N2E2 *gacA* genes can complement a *P. syringae gacA* mutant. We found that in *P. syringae* 485-10-P-3 and 485-10-P-4 *gacA* mutants, *N2C3-gacA* or *N2E2-gacA* restored the fresh weight reduction, root length inhibition, and protease activity (Fig. 5B through D). These data indicate that the restoration of GacA in distantly related *Pseudomonas* complements *in vitro* and *in planta* phenotypes. This restoration of pathogenic traits in GacA-deficient strains illustrates the role of GacA in facilitating the acquisition of virulence traits across the genus *Pseudomonas*.

## DISCUSSION

The GacS/GacA TCS plays a crucial role in determining the lifestyle of *Pseudomonas* species, regulating the delicate balance between pathogenicity and commensalism. This study focuses on the BCM subclade of *P. fluorescens*, particularly the closely related strains *Pseudomonas* spp. N2E2 and N2C3, to uncover the specific role of GacA in facilitating lifestyle transitions rather than simply controlling the expression of individual virulence traits.

Our results reveal that GacA is central to the activation of virulence factors, particularly those acquired through HGT. For instance, in the pathogenic strain N2C3, GacA controls the expression of syringomycin and syringopeptin, key secondary metabolites encoded by genes within a pathogenicity island transferred from other *Pseudomonas* species (Fig. 2). Importantly, GacA is not simply an activator of virulence traits but also functions as a critical mediator of bacterial adaptation. Disruption of GacA in N2C3 abolishes these pathogenic traits, effectively reverting the strain to a non-pathogenic state (Fig. 2A through C). However, GacA itself is highly unstable, with loss-of-function mutants frequently occurring both in culture and in natural environments (14, 28). The tendency for GacA mutations suggests that its lability may be an adaptive feature, allowing bacterial populations to shift between host-associated and free-living niches (28). This aligns with existing literature, which has demonstrated GacA’s role in regulating virulence across various *Pseudomonas* species, including *P. syringae*. In general, soils are nutrient-deprived compared to rich media, where spontaneous GacA mutants often arise. Importantly, when a nutrient-rich environment like a root is present, bacteria may be more likely to toggle and decrease the number of cells that perform certain ecological functions. Several studies show that increased nutrients in host environments correlate with host defense recognition (29, 30). Rather than being a functional flaw, the instability of GacA in rich media may be a strategic adaptation, allowing bacterial populations to modulate their behaviors in response to environmental conditions. This would indicate that syringomycin plays a different role in soil bacteria than in foliar pathogens. It may protect the bacterium from some organisms or inhibit fungi to remove competition in nutrient-poor conditions. Investigating the environmental triggers for this mutation may provide insight into the role of the regulated metabolites in the fitness of the organism in the varied environments they occupy.

The study also highlights GacA’s broader regulatory influence, extending beyond specific pathogenic traits to symbiotic functions essential for plant-microbe interactions. This includes the regulation of protease activity, biofilm formation, and the production of antifungal compounds like DAPG. In N2E2, for example, the loss of GacA results in reduced expression of DAPG and a subsequent decrease in fungal antagonism (Fig. 2D through F), demonstrating GacA’s role in maintaining the beneficial aspects of bacterial-plant interactions. This suggests that GacA’s regulation of antifungal activity is crucial for enhancing plant defense mechanisms, thereby strengthening the symbiotic relationship between bacteria and their plant hosts. Without antimicrobial defense, beneficial strains may be outcompeted or eliminated, disrupting microbial community structure and plant-associated stability (31). DAPG is vital for the biocontrol efficacy of *P. fluorescens* in suppressing soil-borne pathogens like *Fusarium* species (32). Fungal antagonism plays a crucial role in bacterial symbiosis with plants; by regulating DAPG expression, GacA facilitates long-term symbiotic stability within the plant microbiome.

Our heterologous complementation experiments further underscore the versatility and importance of GacA across different strains. When GacA from either N2E2 or N2C3 was reintroduced into gacA-deficient mutants, the mutants regained their lost phenotypic traits, such as root length, fresh weight, and protease activity. Notably, complementation restored symbiotic functions in distantly related *P. syringae* and *P. fluorescens* BCM subclade strains, supporting the idea that GacA functions, even under non-native promoter expression, as a highly conserved regulatory hub. This finding is particularly relevant in the context of HGT, where newly acquired genes require a regulatory framework like GacA to be effectively integrated into the bacterium’s existing genetic and functional landscape.

The ability of GacA to restore symbiotic functions in distantly related *P. syringae* highlights its broad regulatory influence. However, given its tendency for loss-of-function mutations, GacA-mediated regulation may itself be modular and transient, contributing to bacterial phenotypic plasticity. In our study, two spontaneous protease activity-negative mutants were complemented with GacA vectors from N2E2 or N2C3, resulting in partial or complete restoration of their original phenotypes. These data indicate that GacA from *Pseudomonas* can regulate virulence components in distantly related species, suggesting *P. fluorescens* BCM subclade strains are also capable of expressing virulence factors from distantly related strains. As it is likely that SYR/SYP in *Pseudomonas* sp. N2C3 was horizontally acquired (10)*,* these findings suggest that a functional GacA/S pathway in *Pseudomonas* might facilitate these types of HGT events. These results suggest that while GacA plays a role in regulating horizontally acquired virulence genes, its instability may contribute to bacterial adaptability to symbiotic functions rather than strictly maintaining pathogenic traits.

The implications of this study extend beyond the specific strains examined, offering insights into the broader mechanisms of bacterial evolution and adaptation. Lifestyle transitions in bacteria underscore the remarkable genomic plasticity that enables microbes to adapt to changing environments and host interactions. These transitions often involve the acquisition, loss, or regulation of key genes that mediate virulence, symbiosis, or environmental fitness, revealing how subtle genetic shifts can lead to major ecological consequences. Understanding these dynamics not only deepens our knowledge of microbial evolution and host-microbe interactions but also informs strategies for managing plant and human health, predicting pathogen emergence, and engineering beneficial microbiomes for biotechnology.

In agricultural contexts, where *Pseudomonas* species are integral to both plant health and disease, understanding the transient nature of GacA function could provide new strategies for enhancing beneficial plant-microbe interactions while mitigating emerging pathogens. The GacS/GacA TCS, through its regulation of small non-coding RNAs (sRNAs) like RsmY and RsmZ, controls the expression of genes associated with virulence, secondary metabolite production, and stress responses (12, 13). GacA’s role as a dynamic regulatory component, supporting bacterial adaptation in diverse environments, and bridging symbiotic and pathogenic lifestyles. Future research should further explore the balance between regulatory stability and plasticity, particularly in relation to different bacterial lifestyles and environmental cues. This could pave the way for next-generation microbial inoculants tailored to promote plant health while offering an evolutionary conserved toggling mechanism, contributing to more sustainable agricultural practices.

## MATERIALS AND METHODS

### Bacteria growth conditions and selection conditions

All *Pseudomonas* strains were cultured 18–24 h overnight at 28°C using Lysogeny Broth (LB) or agar plates. When appropriate, LB was supplemented with 10% sucrose, 25 µg/mL kanamycin (Kan), 30 µg/mL gentamicin (Gent), and 10 µg/mL nalidixic acid (Nal). To determine the MIC, a plating-based assay was performed. Overnight bacterial cultures were diluted to an OD_600_ of 0.05 in fresh LB medium. Serial 10-fold dilutions of each culture were prepared and spotted (10 µL per spot) onto LB agar plates containing increasing concentrations of the antimicrobial compound (e.g., 0, 0.6, 2, 4, 5, 10, 12.5, 15, 25, and 30 µg/mL). Plates were incubated at 28°C for 24–48 h. The MIC was defined as the lowest concentration at which colony growth changed from 0 µg/mL was observed. All assays were performed in biological triplicate to ensure reproducibility.

### Growth curve analysis

*Pseudomonas* spp. wild-type strains, respective *ΔgacA* mutants*,* and complementation strains were inoculated in LB (24) in a standard flat-bottom 96-well plate at 28°C with a starting from an OD_600 nm_ of 0.02, with hree technical replicates. The bacterial growth was measured spectrophotometrically at 600_nm_ in a continuously shaking plate.

### Strain construction

*Escherichia coli* DH5α was used for the construction and maintenance of plasmids, and *E. coli* SM10 λpir was used for biparental conjugation with *Pseudomonas* spp. Deletion mutants were generated using the two-step allelic exchange method as previously described (33, 34). Deletion constructs for N2E2 ∆*gacA* and N2C3 ∆*gacA* were generated using suitable primers (Table S3).

Genomic DNA was extracted using the Qiagen Puregene kit A. Regions 500–1,000 bp upstream and downstream of the target gene were amplified with High-Fidelity Phusion Polymerase (NEB) and purified using the Qiagen QIAquick PCR Purification Kit. These regions were connected via one-step overlap PCR and digested with the appropriate restriction enzymes (Table S3). The PCR products were then ligated into the pEXG2 suicide vector containing *sacB* and transformed into *E. coli* SM10 λpir. Transformants were selected on LB agar with 30 µg/mL Gm, and the plasmid was transferred to *Pseudomonas* strains via conjugation, integrating into the genome through homologous recombination.

The *gacA* complementation plasmids were created using the pBBR1MCS-2 plasmid as a backbone (35). The N2E2 *gacA* and N2C3 *gacA* genes, along with approximately 200 bp upstream of each gene, were amplified using specific primers (Table S3). These constructs were then electroporated into *Pseudomonas* strains to generate the *gacA* complementation strains (36). Kan was used for selection and maintenance of the plasmid. Wild-type and *∆gacA* strains with an empty vector (EV) were generated similarly by electroporating pBBR1MCS-2 into electrocompetent cells. These cells were prepared by pelleting, washing, and resuspending cultures in 300 mM sucrose. Transformants were selected on and maintained using Kan (25 µg/mL) for *Pseudomonas* spp. *P. syringae* cultures were grown in LB medium at 28°C with shaking (200 rpm). After an initial 2-day growth, 1 mL was transferred to 50 mL of fresh ½-strength LB and grown overnight. This culture was used to inoculate fresh ½-strength LB (50 mL) and grown to an OD_600_ of 0.5–0.6. Exponential-phase cells were washed with 0.5 M sucrose, resuspended in 1 mL of 0.5 M sucrose, and mixed with 5 µg plasmid DNA (100 µL). Electroporation was performed at 25 µF, 200 Ω, and 9.5 kV cm⁻¹ with a ~5 ms time constant. The cells were incubated in LB for 2 h, and transformants were selected on LB plates containing 25 µg/mL kanamycin after 2 days of growth at 28°C (37).

### *In planta* vertical plate disease assays

*Arabidopsis thaliana* Col-0 seeds were sterilized by gas sterilization for 1.5 h. 7 mL of 37% HCl was added to 150 mL of 6% bleach to generate Cl_2_ gas and sterilized seeds were stored in 0.1% sterile phytoagar at 4°C in the dark for 4 days before sowing. ½ Murashige and Skoog (MS) medium plates with 1× MES buffer and 1% sucrose were used to germinate seeds. Plates were then placed vertically in a 23°C growth room with 16 h light, 8 h dark. Five days post-germination, seedlings were then transplanted to no-sucrose ½× MS plates with 1% MES buffer. Roots were inoculated after 5 days of transplanting. All the bacteria were diluted (OD_600nm_ of 0.001) before inoculation (10). The fresh weight of inoculated seedlings was measured 6 days after inoculation.

### *In vitro* assays

#### Competitive fitness

Overnight cultures of wild-type and ∆*gacA* strains of N2C3 and N2E2 were adjusted to equal optical densities (OD_600_ = 0.05), mixed at a 1:1 ratio, and serially diluted in spotted (10 µL) onto LB agar plates supplemented with selective media to distinguish wild-type and ∆*gacA* colonies and subinhibitory concentrations of various antibiotics (5 µg/mL kanamycin; Kan, 1 µg/mL gentamicin; Gent, 5 µg/mL chloramphenicol; Cm, and 10 µg/mL nalidixic acid; Nal). Plates were incubated at 28°C for 24–48 h. Colony-forming units (CFUs) were counted, and the competitive index (CI) was calculated as the ratio of ∆*gacA* to wild-type CFUs at the endpoint, normalized to the input ratio.

#### Antagonism assay

Antagonism assays were performed as previously described in 38*. Aphanomyces euteiches* AE-1 was maintained on potato dextrose agar (PDA) plates, with sub-culturing done by transferring a 5 mm agar plug from an actively growing colony to fresh PDA plates, which were incubated at 25°C for 7–10 days. Bacterial strains were cultured on LB plates and incubated at 28°C for 24 h. Single colonies were scrape inoculated onto antagonism PDA plate. Plates were incubated at 25°C for 7–10 days, and the zone of inhibition around each well was measured to assess the antagonistic effect of the bacterial strains against *A. euteiches*.

#### Biofilm assays

Biofilm assays were performed as previously described in 39. Overnight culture of *Pseudomonas* spp. N2C3 or N2E2 wild type or *ΔgacA*, mutant cells were diluted (OD_600nm_ of 0.1) in M63 medium (1 × M63 salt, 0.2% glucose, 0.5% casamino acids, and 1 mM MgSO_4_) and 100 µL of diluted cultures were incubated at 27°C for 18 h in non-tissue culture-treated 96-well plates. Subsequently, the plate was rinsed, stained with 125 µL of 0.1% crystal violet for 10 min, and dried overnight. The crystal violet was dissolved in 125 µL of 30% acetic acid for 10 min and transferred to a new 96-well, flat-bottom plate. Absorbance was measured at 550 nm using a spectrophotometer (SpectroMax), and all absorbance signals were averaged and normalized against the wild-type values.

#### Protease activity assay

Protease activity was quantified through milk agar clearing as previously described in 31. Briefly, 5 µL of a bacterial suspension was spotted onto the center of each milk agar plate. Plates were incubated at 28°C for 72 h (40). Zones of clearance around the bacterial colonies were measured, indicating the proteolytic activity of the bacteria. Milk agar plates were prepared by adding 20% (vol/vol) sterile skim milk to autoclaved nutrient agar at approximately 50°C, then pouring the mixture into sterile Petri dishes. Plates were allowed to solidify at room temperature.

#### Quorum sensing assay

Bacteria were cultured overnight in LB broth with the appropriate antibiotics at 28°C with shaking at 200 rpm. The reporter strain culture was spread onto plates and allowed to dry. Subsequently, 10 µL of the adjusted signal-producing strain culture and control autoinducer were spotted onto the agar plates. The plates were incubated at 28°C for 48 h. The presence and intensity of purple pigment indicate the level of quorum sensing signal induction (10, 41). Quorum quenching media was supplemented with 0.1% (wt/vol) naringenin (Sigma-Aldrich) (22).

#### Gene expression analysis

Transcripts of genes were quantified via RT-qPCR as described previously (42). RNA was extracted from Kings B liquid cultures using the Qiagen RNAeasy Kit. cDNA was synthesized from normalized RNA using Promega reverse transcriptase. qPCR was performed in triplicate with at least three biological replicates, and gene expression levels were normalized using the ΔCt method with *recA* as the housekeeping gene.

#### Sequencing

To confirm the presence and integrity of the *gacA* gene, we performed Sanger sequencing. Genomic DNA was extracted from bacterial cultures using the Qiagen DNeasy Blood & Tissue Kit. The gacA gene was amplified using high-fidelity Phusion Polymerase (New England Biolabs) and specific primers flanking the *gacA* coding region (Supplementary Data set S1). Purified PCR products were submitted to Azenta for Sanger sequencing. Sequences were compared to reference sequences in the NCBI database using pBLAST restricted to *Pseudomonas* species to confirm the identity and integrity of the *gacA* gene.

#### Phylogenetic gene presence/absence analyzes

Publicly available complete genome sequences of *Pseudomonas* strains N2C3, WCS365, N2E2, and additional representative *Pseudomonas* genomes were used as input. PyParanoid was used to identify orthologous gene clusters across all genomes, as previously described (10). One hundred twenty-two conserved core genes shared across all strains were used for phylogenetic analysis. PyParanoid was used to generate a binary presence/absence matrix for genes of interest, including those associated with DAPG biosynthesis and SYR/SYP toxin production. This matrix was visualized alongside the phylogenetic tree to highlight the distribution of lifestyle-associated genes across strains.

#### Statistics

Statistical analyses were conducted to evaluate differences in bacterial growth and disease symptoms across various experimental treatments. All statistical tests were performed using GraphPad Prism software. The type of analysis applied depended on the experimental design. Student’s *t*-test was used for pairwise comparisons to identify significant differences between two groups. Analysis of variance (ANOVA) was employed for experiments involving multiple groups. For data collected across multiple time points or under repeated measures, a repeated measures ANOVA was used to account for the correlation within subjects over time. Post hoc tests were applied when ANOVA results indicated significance to identify specific group differences. For all tests, statistical significance was defined as *P* < 0.05. Detailed descriptions of the statistical tests used, including their assumptions and the conditions under which they were applied, are provided in the text where results are presented. Results are reported with appropriate measures of central tendency and variability (e.g., mean ± standard deviation of the mean). Graphical representations of the data include error bars to reflect this variability, ensuring clarity and reproducibility in the interpretation of findings.

## Data Availability

All sequencing data supporting the findings of this study are available as part of this published article. The complete GacA sequences are accessible in Table S4.

## SUPPLEMENTAL MATERIAL

Supplemental figures msphere.00277-25-s0001.pdf Figures S1 to S3.

Supplemental tables msphere.00277-25-s0002.xlsx Tables S1 to S3.

Table S4 msphere.00277-25-s0003.xlsx GacA sequence data.

ASM does not own the copyrights to Supplemental Material that may be linked to, or accessed through, an article. The authors have granted ASM a non-exclusive, world-wide license to publish the Supplemental Material files. Please contact the corresponding author directly for reuse.
