# Supplementary material for: GacA regulates symbiosis and mediates lifestyle transitions in Pseudomonas
Source: mSphere. 2025 Sep 8;10(9):e00277-25. doi: 10.1128/msphere.00277-25 (PMC12482163; doi:10.1128/msphere.00277-25)
Supplement: Supplemental figures — Figures S1 to S3. [file msphere.00277-25-s0001.pdf]

A.

```
P_aeruginosa_PAO1                 --------MIKVLVVDDHDLVRTGITRMLADIEGLQVVGQADCGEDCLKLARELKPDVVL 52
P_sp._WCS358                      --------MIRVLVVDDHDLVRTGITRMLADIDGLQVVGEGDSGESALKLARELKPDVVL 52
P_syringae_pv_tomato_DC3000       --------MIKVLVVDDHDLVRTGITRMLADIEGLQVVGQADSGEESLKKARELKPDVVL 52
P_syringae_pv._syringae_B728      MTVARGVCLIKVLVVDDHDLVRTGITRMLADIDGLQVVGQADSGEESLKKARELKPDVVL 60
P_fuscovaginae_SE-1               --------MIRVLVVDDHDLVRTGITRMLADIDGLQVVGQAESGEQALLKTRELKPDVVL 52
P_simiae_WCS417                   --------MIRVLVVDDHDLVRTGITRMLADIDGLQVVGQAESGEESLIKARELKPDVVL 52
P_protegens_Pf-5                  --------MIRVLVVDDHDLVRTGITRMLADIDGLQVVGQAESGEESLLKARELKPDVVL 52
P_sp._UW4                         --------MIRVLVVDDHDLVRTGITRMLADIDGLQVVGQAESGEESLLKARELKPDVVL 52
P_brassicacearum_DF41             --------MIRVLVVDDHDLVRTGITRMLADIDGLQVVGQAESGEEALIKARELKPDVVL 52
P_fluorescens_FW300-N2C3          ---------------------------MLADIDGLQVVGQAESGEESLIKARELKPDVVL 33
P_fluorescens_FW300-N2E2          ---------------------------MLADIDGLQVVGQAESGEESLIKARELKPDVVL 33
P_brassicacearum_NFM421           --------MIRVLVVDDHDLVRTGITRMLADIDGLQVVGQAESGEESLIKARELKPDVVL 52
                                                             *****:******:.:.**..*  :*********

P_aeruginosa_PAO1                 MDVKMPGIGGLEATRKLLRSQPDIKVVVVTVCEEDPFPTRLMQAGAAGYMTKGAGLEEMV 112
P_sp._WCS358                      MDVKMPGIGGLEATRKLLRSHPDIKVVAVTVCEEDPFPTRLLQAGAAGYLTKGAGLDEMV 112
P_syringae_pv_tomato_DC3000       MDVKMPGIGGLEATRKMLRSHPDIKVVAVTACEEDPFPTRLLQAGAAGYMTKGAGLAEMV 112
P_syringae_pv._syringae_B728      MDVKMPGIGGLEATRKMLRSHPDIKVVAVTVCEEDPFPTRLLQAGAAGYMTKGAGLAEMV 120
P_fuscovaginae_SE-1               MDVKMPGIGGLEATRKLLRSHPDIKVVAVTVCEEDPFPTRLMQAGASGYLTKGAGLAEMV 112
P_simiae_WCS417                   MDVKMPGIGGLGATTKLLRSHPDIKVVVVTVCEEDPFPTRLLQAGAAGYLTKGAGLAEMV 112
P_protegens_Pf-5                  MDVKMPGIGGLEATRKLLRSHPDIKVVAVTVCEEDPFPTRLLQAGAAGYLTKGAGLNEMV 112
P_sp._UW4                         MDVKMPGIGGLEATRKLLRSHPDIKVVAVTVCEEDPFPTRLLQAGAAGYLTKGAGLPEMV 112
P_brassicacearum_DF41             MDVKMPGIGGLEATRKLLRSHPDIKVVAVTVCEEDPFPTRLLQAGAAGYLTKGAGLNEMV 112
P_fluorescens_FW300-N2C3          MDVKMPGIGGLEATRKLLRSHPDIKVVAVTVCEEDPFPTRLLQAGAAGYLTKGAGLNEMV 93
P_fluorescens_FW300-N2E2          MDVKMPGIGGLEATRKLLRSHPDIKVVAVTVCEEDPFPTRLLQAGAAGYLTKGAGLNEMV 93
P_brassicacearum_NFM421           MDVKMPGIGGLEATRKLLRSHPDIKVVAVTVCEEDPFPTRLLQAGAAGYLTKGAGLNEMV 112
                                  *********** ** *:***:******.**.**********:****:**:****** ***

P_aeruginosa_PAO1                 QAIRQVFAGQRYISPQIAQQLALKSFQPQQHDSPFDSLSEREIQIALMIANCHKVQSISD 172
P_sp._WCS358                      QAIRLAFAGQRYISPQIAQQLALKSFQPQ--GSPFDALSEREIQIALMIVGCQKVQIISD 170
P_syringae_pv_tomato_DC3000       QAIRLVFAGQRYISPQIAQQLALKSFQPQVNNSPFDLLSEREIQIALMIVGCQKVQTISD 172
P_syringae_pv._syringae_B728      QAIRLVFAGQRYISPQIAQQLALKSFQPQVNNSPFDLLSEREIQIALMIVGCQKVQTISD 180
P_fuscovaginae_SE-1               QAIRLVFAGQRYISPQIAQQMALKSFQPA-SESPFDALSEREIQIALMIVGCQKVQIISD 171
P_simiae_WCS417                   QAIRLVFAGQRYISPQIAQQLAIKSFQPV-SDSPFDVLSEREIQIALMIVGCQKVQTISD 171
P_protegens_Pf-5                  QAIRLVFAGQRYISPQIAQQLVFKSFQPS-SDSPFDALSEREIQIALMIVGCQKVQIISD 171
P_sp._UW4                         QAIRLVFAGQRYISPQIAQQLAIKSFQPT-NDSPFDALSEREIQIALMIVGCQKVQIISD 171
P_brassicacearum_DF41             QAIRLVFAGQRYISPQIAQQLAIKSFQPT-NDSPFDALSEREIQIALMIVGCQKVQIISD 171
P_fluorescens_FW300-N2C3          QAIRLVFAGQRYISPQIAQQLAIKSFQPT-NDSPFDALSEREIQIALMIVGCQKVQIISD 152
P_fluorescens_FW300-N2E2          QAIRLVFAGQRYISPQIAQQLAIKSFQPT-NDSPFDALSEREIQIALMIVGCQKVQIISD 152
P_brassicacearum_NFM421           QAIRLVFAGQRYISPQIAQQLAIKSFQPT-NDSPFDALSEREIQIALMIVGCQKVQIISD 171
                                  **** .**************:.:*****    **** ************..*:*** ***

P_aeruginosa_PAO1                 KLCLSPKTVNTYRYRIFEKLSITSDVELALLAVRHGMVDAAS 214
P_sp._WCS358                      KLCLSPKTVNTYRYRIFEKLSVTSDVELTLLAVRHGMVDASL 212
P_syringae_pv_tomato_DC3000       KLCLSPKTVNTYRYRIFEKLSISSDVELALLAVRHGMVDASA 214
P_syringae_pv._syringae_B728      KLCLSPKTVNTYRYRIFEKLSISSDVELALLAVRHGMVDASA 222
P_fuscovaginae_SE-1               KLCLSPKTVNTYRYRIFEKLSVSSDVELTLLAVRHGMVDAG- 212
P_simiae_WCS417                   KLCLSPKTVNTYRYRIFEKLAISSDVELTLLAVRHGMVDASA 213
P_protegens_Pf-5                  KLCLSPKTVNTYRYRIFEKLSISSDVELTLLAVRHGMVDASL 213
P_sp._UW4                         KLCLSPKTVNTYRYRIFEKLSISSDVELTLLAVRHGMVDASL 213
P_brassicacearum_DF41             KLCLSPKTVNTYRYRIFEKLSISSDVELTLLAVRHGMVDASA 213
P_fluorescens_FW300-N2C3          KLCLSPKTVNTYRYRIFEKLSISSDVELTLLAVRHGMVDASA 194
P_fluorescens_FW300-N2E2          KLCLSPKTVNTYRYRIFEKLSISSDVELTLLAVRHGMVDASA 194
P_brassicacearum_NFM421           KLCLSPKTVNTYRYRIFEKLSISSDVELTLLAVRHGMVDASA 213
                                  ********************:::*****:***********.
```

B.

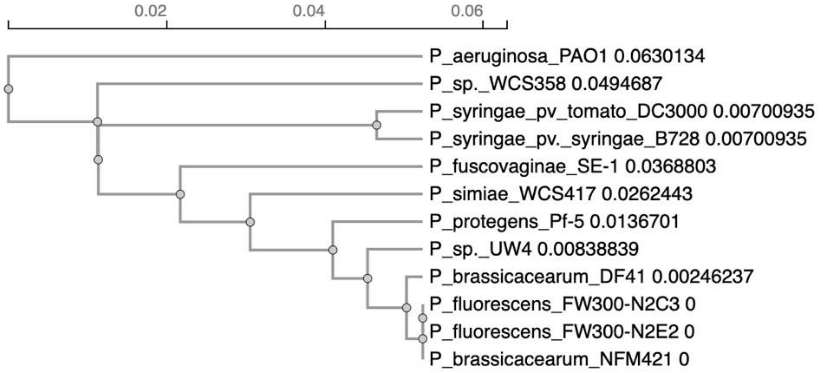

Figure S1. Alignment of GacA from strains across the genus *Pseudomonas* shows a high degree of conservation of GacA sequences. A. Clustal omega alignment of predicted GacA amino acid sequences. B. Neighbor joining tree showing relatedness of GacA sequences.

A.

```
P_aeruginosa_PAO1                ----MFKDLGIKGRVLLLTLLPTSLLAMVLGGYFTWVQLSDMRAQLIERGQLIAEQLAPL 56
P_sp._WCS358                     ----MLDRLGIRSRVLLLALLPAGLMALVLGSYFTWLQQSDLRTQLLQRGKMIAEQLAPL 56
P_syringae_pv_tomato_DC3000      ----MLTKLGIKGRELLLTILPASLMAAMLGGYFTWMQLSELQSQLLLRGEMIAQDLAPL 56
P_syringae_pv._syringae_B728     ----MLTKLGIKGRVLLLTILPASLMAAMLGGYFTWMQLSELQSQLLQRGEMIAQDLAPL 56
P_fuscovaginae_SE-1              ----MLKNLGIKGRVLLLTLLPTTLMALVLGGYFTWLQQSELQSQLLQRGEMIAEQLAPL 56
P_simiae_WCS417                  ----MLTRMGIKGRVLLLTLLPTSLMASLLGGYFTWMQLSELQTQLLQRGEMIAEQLAPL 56
P_protegens_Pf-5                 ----MLKKLGIKGRVLLLTLLPTSLMALVLGGYFTWMQLSDLQTQLLQRGEMIAEQLASL 56
P_sp._UW4                        ----MLKKLGIKGRVLLLTLLPTSLMALVLGGYFTWMQQADLQTQLMQRGEMIAEQLAPL 56
P_brassicacearum_DF41            ----MLKKLGIKGRVLLLTLLPTSLMALVLGGYFTWMQQSDLHAQLLQRGEMIAEQLAPL 56
P_fluorescens_FW300-N2E2         ----MLKKLGIKGRVLLLTLLPTSLMALVLGGYFTWMQQSELHAQLLQRGEMIAEQLAPL 56
P_fluorescens_FW300-N2C3         ----MLKKLGIKGRVLLLTLLPTSLMALVLGGYFTWMQQSDLHAQLLQRGEMIAEQLAPL 56
P_brassicacearum_NFM421          ----MLKKLGIKGRVLLLTLLPTSLMALVLGGYFTWMQQSDLHAQLLQRGEMIAEQLAPL 56
                                     *:  :**:.* ***::**: *:* :**.****:* :::::**: **::**::** *

P_aeruginosa_PAO1                AATALARKDTAVLNRIANEALDQPDVRAVTFLDARQERLAHAGPSMLTVAPAG-DASHLS 115
P_sp._WCS358                     AAPAMARLAPAQLERIAAQTLEQADVRAVAFLAPDRTRLAHAGPSMLNQPPSGGTGTQLL 116
P_syringae_pv_tomato_DC3000      SASALGRKDKVLLSRIATQTLEQPDVRAVSFLDVDRSPLAHAGPTMISPTPIG-NSSQLL 115
P_syringae_pv._syringae_B728     AANALGRKDKVLLSRIATQTLEQTDVRAVSFLDTDRTVLAHAGPTMISPSPIG-SGSQLL 115
P_fuscovaginae_SE-1              VAPALSRNDSEQLERVATQALEQTDVRAVSFLGANREQLAHAGPSMLNQPPTG-NSSHML 115
P_simiae_WCS417                  VAPALSARNTELLERIATQSLEQPDVRAVSFLSPERAPLAHAGPTMLNQPPVG-NSSHLL 115
P_protegens_Pf-5                 VAPAMGNHNTQMLERIATQALEQQDVRAVSLLAPDRSLLAHAGPSMLNPPPAG-NSSHMM 115
P_sp._UW4                        AAPAMGHQDNELLERIATQALEQTDVRAVTFLAPDRTLLAHAGPTMLNQVPSG-SGMQML 115
P_brassicacearum_DF41            VAPAMSRQDTELLERVATQSLEQTDVRAVTFLAPDRTSLAHAGPTMLNRAPEG-NSAQLL 115
P_fluorescens_FW300-N2E2         VAPAMSRQDIDLLERIATQSLEQADVRAVTFLAPDRVPLAHAGPTMLNRAPEG-NSTQLL 115
P_fluorescens_FW300-N2C3         VAPAMSRQDTDLLERIATQSLEQADVRAVTFLAPDRAPLAHAGPTMLNRAPEG-NSAQLL 115
P_brassicacearum_NFM421          VAPAMSRQDTDLLERIATQSLEQADVRAVTFLAPDRIPLAHAGPTMLNRAPEG-NSTQLL 115
                                  * *:.      *.*:* ::*:* *****::*   :  ******:*:.  * *  . ::

P_aeruginosa_PAO1                MSTELDTTHFLLPVLGRHHSLSGATEPD-DERVLGWVELELSHHGTLLRGYRSLFTSLLL 174
P_sp._WCS358                     QRTGNDATRYLMPVFGHHRDLATDAVPAEAERLLGWVEIELSHDGTLLRGYRNLFTSLLL 176
P_syringae_pv_tomato_DC3000      NSTGTDATRYLLPVFGNQRHLTSPIIPAEADTLMGWVDVEISHNGTLLRGYRSLFASLLL 175
P_syringae_pv._syringae_B728     SSTGTDATRYLLPVFGSQRHLTSPIIPAEADTLLGWVELEISHNGTLLRGYRSLFASLLL 175
P_fuscovaginae_SE-1              QRSGNDATRYLLPVFGRHRGMAGEPVPDEADRLLGWVELELSHNGMLLRGYRSLFASLLL 175
P_simiae_WCS417                  QRTGNDATRYLLPVFGRHRNLAGELIPDEADRLLGWVEVELSHNGMLLRGYRSLFASLLL 175
P_protegens_Pf-5                 QRSGSDATRYQLPVFGRHRNLAGDLIPDESDRLLGWVELELSHSGMLLRGYRSLFASLLL 175
P_sp._UW4                        RRSGNDATRYLLPVFGKHRNLAGDVISEEADRLLGWVELELSHNSMLLRGYRSLFASLML 175
P_brassicacearum_DF41            QRTGNDATRYLLPVFGKHRNLAGELIPEESDRLLGWVELELSHSGMLLRGYRSLFASLLL 175
P_fluorescens_FW300-N2E2         QRTGNDATRYLLPVFGKHRNLAGELIPEEADRLLGWVELELSHSGMLLRGYRSLFASLLL 175
P_fluorescens_FW300-N2C3         QRTGNDATRYLLPVFGKHRNLAGELIPEEADRLLGWVELELSHSGMLLRGYRSLFASLLL 175
P_brassicacearum_NFM421          QRTGNDATRYLLPVFGKHRNLAGELIPEEADRLLGWVELELSHSGMLLRGYRSLFASLLL 175
                                   :  *:*:: :**:* :: ::        : ::***::*:** . ******.**:**:*

P_aeruginosa_PAO1                IAAGLGVTALLALRMSRAINAPLELISQGVAQLKEGRMETRLPPMGSNELDELASGINRM 234
P_sp._WCS358                     ILACLALSGLLALRMSRTINDPIKRIKHTVNQLKDGHLDERLPAMGSHELDELARGINRM 236
P_syringae_pv_tomato_DC3000      IFTGLAITAVVAVRLSRTINGPMSQIKQAVSQLKDGNLETRLPPLGSRELDELASGINRM 235
P_syringae_pv._syringae_B728     ILTGLAFTATLAVRMSRTINGPMSQIKQAVSQLKDGNLETRLPPLGSRELDELASGINRM 235
P_fuscovaginae_SE-1              IATGLLGTALLALRMSRTINTPISQIRQAVAQLKDGNLETRLPALGSQELDQLASGINRM 235
P_simiae_WCS417                  IAIGLICTAALALRISRTINSPIGQIKQAVAQLKDGNLETRLPPLGSQELDQLASGINRM 235
P_protegens_Pf-5                 IAAGLAGTALLAVRMGRTINNPLTQIKQAVAQLKDGNLETRLPPLGSQELDELASGINRM 235
P_sp._UW4                        IAAGLTGAALLALRMGRTINRPLSQIKQAVAQLKDGHLETRLPPLGSQELDELASGINRM 235
P_brassicacearum_DF41            ITAGLGLTALLALRMGRTINRPLSQIKQAVAQLKDGHLETRLPPLGSQELDELASGINRM 235
P_fluorescens_FW300-N2E2         IAAGLCLTGLLALRMGRTINRPLSQIKQAVAQLKDGHLETRLPPLGSQELDELASGINRM 235
P_fluorescens_FW300-N2C3         IAAGLCLTALLALRMGRTINRPLSQIKQAVAQLKDGHLETRLPPLGSQELDELASGINRM 235
P_brassicacearum_NFM421          IAAGLCLTGLLALRMGRTINRPLSQIKQAVAQLKDGHLETRLPPLGSQELDELASGINRM 235
                                 *   *  :. :*:*:.*:** *:  * : * ***:*.:: *** :**.***:** *****

P_aeruginosa_PAO1                AETLQSAQEEMQHNIDQATEDVRQNLETIEIQNIELDLARKEALEASRIKSEFLANMSHE 294
P_sp._WCS358                     AETLHSAHEELQHSIDQATEDVRQNLETIEIQNIELDMARKEALEASRIKSEFLANMSHE 296
P_syringae_pv_tomato_DC3000      AATLQNAQEELQMSIDQATEDVRQNLETIEIQNIELDLARKEALEASRIKSEFLANMSHE 295
P_syringae_pv._syringae_B728     AATLQNAQEELQLSIDQATEDVRQNLETIEIQNIELDLARKEALEASRIKSEFLANMSHE 295
P_fuscovaginae_SE-1              AETLQNAQEELQHSIDQATEDVRQNLETIEIQNIELDLARKEALEASRIKSEFLANMSHE 295
P_simiae_WCS417                  AETLQNAQEELQHSIDQATEDVRQNLETIEIQNIELDLARKEALEASRIKSEFLANMSHE 295
P_protegens_Pf-5                 ASTLQNAQEELQHSIDQATEDVRQNLETIEIQNIELDLARKEALEASRIKSEFLANMSHE 295
P_sp._UW4                        AGTLQNAQEELQHSIDQATEDVRQNLETIEIQNIELDLARKEALEASRIKSEFLANMSHE 295
P_brassicacearum_DF41            AGTLQNAQEELQHSIDQATEDVRQNLETIEIQNIELDLARKEALEASRIKSEFLANMSHE 295
P_fluorescens_FW300-N2E2         ASTLQNAQEELQHSIDQATEDVRQNLETIEIQNIELDLARKEALEASRIKSEFLANMSHE 295
P_fluorescens_FW300-N2C3         ASTLQNAQEELQHSIDQATEDVRQNLETIEIQNIELDLARKEALEASRIKSEFLANMSHE 295
P_brassicacearum_NFM421          ASTLQNAQEELQHSIDQATEDVRQNLETIEIQNIELDLARKEALEASRIKSEFLANMSHE 295
                                 * **:.*:**:* .***********************:**********************

P_aeruginosa_PAO1                IRTPLNGILGFTNLLQKSELSPRQQDYLTTIQKSAESLLGIINEILDFSKIEAGKLVLEN 354
P_sp._WCS358                     IRTPLNGILGFTHLLQKSELTPRQLDYLSTIEKSADNLLGIINEILDFSKIEAGKLVLDS 356
P_syringae_pv_tomato_DC3000      IRTPLNGILGFTHLLQKSELTPRQFDYLATIEKSADNLLSIINEILDFSKIEAGKLVLDN 355
P_syringae_pv._syringae_B728     IRTPLNGILGFTHLLQKSELTPRQFDYLGTIEKSADNLLSIINEILDFSKIEAGKLVLDN 355
P_fuscovaginae_SE-1              IRTPLNGILGFTHLLQKSELTPRQLDYLSTIEKSADSLLGIINEILDFSKIEAGKLVLDS 355
P_simiae_WCS417                  IRTPLNGILGFTHLLQKSELTPRQLDYLGTIEKSADNLLGIINEILDFSKIEAGKLVLDS 355
P_protegens_Pf-5                 IRTPLNGILGFTHLLQKSELTPRQLDYLGTIEKSADSLLGIINEILDFSKIEAGKLVLDS 355
P_sp._UW4                        IRTPLNGILGFTHLLQKSELTPRQLDYLGTIEKSADSLLGIINEILDFSKIEAGKLVLDH 355
P_brassicacearum_DF41            IRTPLNGILGFTHLLQKSELTPRQLDYLGTIEKSADSLLGIINEILDFSKIEAGKLVLDN 355
P_fluorescens_FW300-N2E2         IRTPLNGILGFTHLLQKSELTPRQLDYLGTIEKSADSLLGIINEILDFSKIEAGKLVLDS 355
```

```
P_fluorescens_FW300-N2C3         IRTPLNGILGFTHLLQKSELTPRQLDYLGTIEKSADSLLGIINEILDFSKIEAGKLVLDS 355
P_brassicacearum_NFM421          IRTPLNGILGFTHLLQKSELTPRQLDYLGTIEKSADSLLGIINEILDFSKIEAGKLVLDS 355
                                 ************:*******:*** *** **:***:.**.******************:

P_aeruginosa_PAO1                LPFNLRDLIQDALTMLAPAAHEKQLELVSLVYRDTPIQLQGDPQRLKQILTNLVGNAIKF 414
P_sp._WCS358                     IPFNLRDLVQDTLTILAPAAHAKQLELLSLIYRDTPSSLVGDPLRLKQILTNLVSNAIKF 416
P_syringae_pv_tomato_DC3000      IPFNLRDLLQDTLTILAPAAHAKHLELVSLVYRDTPLALSGDPLRLRQILTNLVSNAIKF 415
P_syringae_pv._syringae_B728     IPFNLRDLLQDTLTILAPAAHAKQLELVSLVYRDTPLALSGDPLRLRQILTNLVSNAIKF 415
P_fuscovaginae_SE-1              IPFNLRDLLQDTLTILAPAAHAKQLELVSLVYRDTPLSLIGDPLRLKQILTNLVSNAIKF 415
P_simiae_WCS417                  IPFNLRDLLQDTLTILAPAAHAKQLELVSLVYRDTPLALVGDPLRLKQILTNLISNAIKF 415
P_protegens_Pf-5                 IPFNLRDLLQDTLTILAPAAHAKQLELVSLVYRDTPLSLVGDPLRLKQILTNLVSNAIKF 415
P_sp._UW4                        IPFNLRDLLQDTLTILAPAAHAKQLELVSLVYRDTPLSLVGDPLRLKQILTNLVSNAIKF 415
P_brassicacearum_DF41            IPFNLRDLLQDTLTILAPAAHAKQLELVSLVYRDTPLSLVGDPLRLKQILTNLVSNAIKF 415
P_fluorescens_FW300-N2E2         IPFNLRDLLQDTLTILAPAAHAKQLELVSLVYRDTPLSLVGDPLRLKQILTNLVSNAIKF 415
P_fluorescens_FW300-N2C3         IPFNLRDLLQDTLTILAPAAHAKQLELVSLVYRDTPLSLVGDPLRLKQILTNLVSNAIKF 415
P_brassicacearum_NFM421          IPFNLRDLLQDTLTILAPAAHAKQLELVSLVYRDTPLSLVGDPLRLKQILTNLVSNAIKF 415
                                 :*******:**:**:****** *:***:**:*****  * *** **:******:.*****

P_aeruginosa_PAO1                TQGGTVAVRAMLEDESDDRAQLRISVQDTGIGLSEEDQQALFKAFSQADNSLSRQAGGTG 474
P_sp._WCS358                     TREGTIVVRAMLEDEREDSAQLRISVQDTGIGLSPQDVRTLFQAFSQADNSLARQPGGTG 476
P_syringae_pv_tomato_DC3000      TREGTIVARAMLEDETEEHAQLRISVQDTGIGLSSQDVRALFQAFSQADNSLSRQPGGTG 475
P_syringae_pv._syringae_B728     TREGTIVARAMLEDETEEHAQLRISVQDTGIGLSSQDVRALFQAFSQADNSLSRQPGGTG 475
P_fuscovaginae_SE-1              TREGTIVARAMLEEEHEDSVQLRISVQDTGIGLSSQDVRALFQAFSQADNSLSRQPGGIG 475
P_simiae_WCS417                  TREGTIVARAMIEDEQEDSVQLRISVQDTGIGLSSQDVRALFQAFSQADNSLSRQPGGTG 475
P_protegens_Pf-5                 TREGTIVARAMLEEEHEDSVQLRISIQDTGIGLSNQDVRALFQAFSQADNSLSRQPGGTG 475
P_sp._UW4                        TREGTIVARAMLEEEHEDSVQLRISIQDTGIGLSNQDVRALFQAFSQADNSLSRQPGGTG 475
P_brassicacearum_DF41            TREGTIVARAMLEDEHEDSVQLRISIQDTGIGLSNQDVRALFQAFSQADNSLSRQPGGTG 475
P_fluorescens_FW300-N2E2         TREGTIVARAMLEDEHEDSVQLRISIQDTGIGLSNQDVRALFQAFSQADNSLSRQPGGTG 475
P_fluorescens_FW300-N2C3         TREGTIVARAMLEDEHEDSVQLRISIQDTGIGLSNQDVRALFQAFSQADNSLSRQPGGTG 475
P_brassicacearum_NFM421          TREGTIVARAMLEDEHEDSVQLRISIQDTGIGLSNQDVRALFQAFSQADNSLSRQPGGTG 475
                                 *: **:..***:*:* :: .*****:******** :* ::**:*********:** ** *

P_aeruginosa_PAO1                LGLVISKRLIEQMGGEIGVDSTPGEGAEFWISLSLPKSRDDNEEPGASWAAGQRVALLEP 534
P_sp._WCS358                     LGLVISKRLIEQMGGEIGVDSTPGEGSQFWISLNLPKAHDDAEDLPLQPLLGRRAAIVDS 536
P_syringae_pv_tomato_DC3000      LGLVISKRLIEQMGGEIGVDSTPGEGSEFWISLKLPKAREDKEESLNLPLEGLRAAVLEH 535
P_syringae_pv._syringae_B728     LGLVISKRLIEQMGGEIGVDSTPGEGSEFWISLKLPKAREDKEESLNIPLGGLRAAVLEH 535
P_fuscovaginae_SE-1              LGLVISKRLIEQMGGEIGVDSTPGEGSEFWISLSLPKTRDDIEDLPAAPLLGRRVAVLEH 535
P_simiae_WCS417                  LGLVISKRLIEQMGGEIGVDSTPGEGSEFWISLNLPKTRDDVEDLPSAPLLGHRVAVLEN 535
P_protegens_Pf-5                 LGLVISKRLIEQMGGEIGVDSTPGEGSEFWISLNLPKTRDDAEDLPGPPLLGRRVAVLEN 535
P_sp._UW4                        LGLVISKRLIEQMGGEIGVDSTPGEGSEFWISLSLPKARDDAEDLPAAPLLGRRVAVLEN 535
P_brassicacearum_DF41            LGLVISKRLVEQMGGEIGVDSTPGEGSEFWISLRLPKTRDDAEDLPGPPLLGRRVAVLEN 535
P_fluorescens_FW300-N2E2         LGLVISKRLVEQMGGEIGVDSTPGEGSEFWISLRLPKTRDDAEDLPGPPLLGRRVAVLEN 535
P_fluorescens_FW300-N2C3         LGLVISKRLVEQMGGEIGVDSTPGEGSEFWISLRLPKTRDDAEDLPGPPLLGRRVAVLEN 535
P_brassicacearum_NFM421          LGLVISKRLVEQMGGEIGVDSTPGEGSEFWISLRLPKTRDDAEDLPGPPLLGRRVAVLEN 535
                                 *********:****************::***** ***:::* *:        * *.*:::

P_aeruginosa_PAO1                QELTRRSLHHQLTDFGLEVSEFADLDSLQESLRNPPPGQLPISLAVLGVSAAIHPPEELS 594
P_sp._WCS358                     HELARQALEHQLEDCGLSVSLFASFDQLLHAVQAAHQAGHPFDAVLGANLGNLTPEQLG 596
P_syringae_pv_tomato_DC3000      HDLARQALEHQLEDCGLHTVVFTNLENLLNGVTAAHETPAAIDLAVLGVTTLEISPERLR 595
P_syringae_pv._syringae_B728     HDLARQALEHQLEDCGLQTIVFNNLENLLNGVTAAHETPAAIDLAVLGVTALEISPERLR 595
P_fuscovaginae_SE-1              HELARQALQHQLEDCGLEVIPFNSLPGLANGVTGAYQTEQAIDLAVVGVTAQDMPPERLN 595
P_simiae_WCS417                  HELARQALQHQLEDCGLEVTPFNSLESLTNGITSAHQTEQAIDLAVLGVTANDIPPERLN 595
P_protegens_Pf-5                 HELARQALQHQLEDCGLEVTPFNTLEALTNGITGVHQSEQAIDLAVLGITTNDMSPERLS 595
P_sp._UW4                        HELARQALQHQLEDCGLSTTPFNTLESLTNGVTAAHQTDQAIDLAVLGITSNDMPPERLN 595
P_brassicacearum_DF41            HELARQALQHQLEDCGLQVTPFNTLESLTNGVTVAHQTEQAIDLAVLGITSNDMPPERLN 595
P_fluorescens_FW300-N2E2         HELARQALQHQLEDCGLQVTPFNTLESLTNGITIAHQTDQAIDLAVLGITSNDMPPERLN 595
P_fluorescens_FW300-N2C3         HELARQALQHQLEDCGLQVTPFNTLESLTNGITVAHQTDQAIDLAVLGITSNDMPPERLN 595
P_brassicacearum_NFM421          HELARQALQHQLEDCGLQVTPFNTLESLTNGITVAHQTDQAIDLAVLGITSNDMPPERLN 595
                                 ::*:*::*.*** * **  .  *  :  * ..:         :.:**:* .     **.*

P_aeruginosa_PAO1                QSFWEFERLGCKTLVLCPTTEQAQYHATLPD--EQVEAKPACTRKLQRKLQELLQVRPTR 652
P_sp._WCS358                     EYHLHLERYQCQCVVLCPTTEQALYHPYLPNGHGQLLSKPTCTRKLRRLLLELVQPRRPL 656
P_syringae_pv_tomato_DC3000      QHIWDLENLNCKVMVLCPTTEYALFQLAVHDVYTQLQAKPACTRKLQKALSELIAPRAIR 655
P_syringae_pv._syringae_B728     QHIWDLENLNCKVMVLCPTTEHALFQLAVHDVYTQLQAKPACTRKLQKALSELIAPRAVR 655
P_fuscovaginae_SE-1              QHIWDLEHLGCRVLVLCPTTEQTLFHLSVPNPHNQLQAKPACTRKLRRTLSDLIAPRPAR 655
P_simiae_WCS417                  QHLWDLEHLGCKVLVLCPTTEQMLFNQSVPNPNSQLQAKPACTRKLRRALSDLISPRPSR 655
P_protegens_Pf-5                 QHIWDLEHLGCKVLVLCPTTEQTLFHLSVPNPHSQLQAKPACTRKLRRALSDLVTPRRAR 655
P_sp._UW4                        QHIWDLEHLGCKVLVLCPTTEQTLFHLSVPNPHSQLQAKPACTRKLRRSLSDLVSPRPMR 655
P_brassicacearum_DF41            QHIWDLEHLGCKVLVLCPTTEQTLYHLSVPNPHSQLQAKPACTRKLRRSLSDLVNPRPTR 655
P_fluorescens_FW300-N2E2         QHIWDLEHLGCKVLVLCPTTEQTLYHLSVPNPHSQLQAKPACTRKLRRALSDLVNPRPTR 655
P_fluorescens_FW300-N2C3         QHIWDLEHLGCKVLVLCPTTEQTLYHLSVPNPHSQLQAKPACTRKLRRALSDLVNPRPPR 655
P_brassicacearum_NFM421          QHIWDLEHLGCKVLVLCPTTEQTLYHLSVPNPHSQLQAKPACTRKLRRALSDLVNPRPTR 655
                                 :   .:*.  *: :*******    ::  : :   *: :**:*****:: * :*:  *

P_aeruginosa_PAO1                SDKPHAMVSGRPPRLLCVDDNPANLLLVQTLLSDLGAQVTAVDSGYAALEVVQRERFDLV 712
P_sp._WCS358                     GE-PNVHSGQRQPKILCVDDNAANLLLVQTLLEDLGAEVLAVDNGYAAVQAVQDEPFDLV 715
P_syringae_pv_tomato_DC3000      AD-IAPPLSSRAPRVLCVDDNPANLLLVQTLLEDMGAEVMAVEGGYAAVNAVQQEAFDLV 714
P_syringae_pv._syringae_B728     AD-IGPPLSSRAPRVLCVDDNPANLLLVQTLLEDMGAEVVAVEGGYAAVNAVQQEAFDLV 714
P_fuscovaginae_SE-1              LE-PSEPLGSRAPRVLCVDDNPANLLLVQTLLEDMGAEVLAVDSGYAAIDAVQQASFDLV 714
P_simiae_WCS417                  SE-PGEPLSSRAPRVLCVDDNPANLLLVQTLLEDMGARVRAVESGYAAIDAVKQDTFDLV 714
P_protegens_Pf-5                 SE-PEETLSSRAPRVLCVDDNPANLLLIQTLLEDMGAKVLAVDNGYAALNAIQTEPFDLV 714
```

```
P_sp._UW4                         SE-PGEPVSSRAPKVLCVDDNPANLMLVQTLLEDMGAKVLAVESGYAAVKAVQNETFDLV 714
P_brassicacearum_DF41             SE-PHEPVASRAPKVLCVDDNPANLLLVQTLLEDMGAKVLAVESGYAAVKAVQKETFDLV 714
P_fluorescens_FW300-N2E2          SD-PHEPVSSRAPKVLCVDDNPANLLLVQTLLEDMGAKVLAVESGYAAVKAVQKETFDLV 714
P_fluorescens_FW300-N2C3          SE-PSEPVSSRAPKVLCVDDNPANLLLVQTLLEDMGAKVLAVESGYAAVKAVQKETFDLV 714
P_brassicacearum_NFM421           SE-PHEPVSSRAPKVLCVDDNPANLLLVQTLLEDMGAKVLAVESGYAAVKAVQKETFDLV 714
                                   :      . * *::****** ***:*:****.*:**.* **:.****:..::   ****

P_aeruginosa_PAO1                 FMDVQMPGMDGRQATEAIRRWEAEREVSPVPVIALTAHALSNEKRALLQAGMDDYLTKPI 772
P_sp._WCS358                      LMDVQMPGMDGRACTEQIRLWENAQNGSPLPIVALTAHAMANEKRALLHAGMDDYLTKPI 775
P_syringae_pv_tomato_DC3000       LMDVQMPGMDGRQATEAIRAWEAERGQSSLPIVALTAHAMANEKRSLLQSGMDDYLTKPI 774
P_syringae_pv._syringae_B728      LMDVQMPGMDGRQATEAIRAWEAERNQSSLPIVALTAHAMANEKRSLLQSGMDDYLTKPI 774
P_fuscovaginae_SE-1               LMDVQMPGMDGRQATEAIRQWENERQSAALPIVALTAHAMANEKRALLQSGMDDYLTKPI 774
P_simiae_WCS417                   LMDVQMPGMDGRQSTEAIRQWESERSGTPLPVVALTAHAMANEKRALLQSGMDDYLTKPI 774
P_protegens_Pf-5                  MMDVQMPGMDGRQSTEAIRQWESERHGTPLPIVALTAHAMANEKRALLQSGMDDYLTKPI 774
P_sp._UW4                         LMDVQMPGMDGRQSTEAIRQWESERHCTPLPIVALTAHAMANEKRALLQSGMDDYLTKPI 774
P_brassicacearum_DF41             LMDVQMPGMDGRQSTEAIRQWESERHCTPLPIVALTAHAMANEKRALLQSGMDDYLTKPI 774
P_fluorescens_FW300-N2E2          LMDVQMPGMDGRQSTEAVRQWESERHCTPLPIVALTAHAMANEKRALLQSGMDDYLTKPI 774
P_fluorescens_FW300-N2C3          LMDVQMPGMDGRQSTEAIRQWESERHCTPLPIVALTAHAMANEKRALLQSGMDDYLTKPI 774
P_brassicacearum_NFM421           LMDVQMPGMDGRQSTEAIRQWESERHCTPLPIVALTAHAMANEKRALLQSGMDDYLTKPI 774
                                  :*********** .** :* **  :  : :*::******::****:**::**********

P_aeruginosa_PAO1                 DEQQLAQVVLKWTGLSLGQSLASMS--RAPQLGQLSVLDPEEGLRLAAGKADLAADMLAM 830
P_sp._WCS358                      SERQLAQVVMKWTGLSLGMPQQERLAEQLANSDELKVLDPEEGLRLAAGKPDLAADMLAM 835
P_syringae_pv_tomato_DC3000       SERQLAQVVLKWSGLALRNPAPERQNESLEAHVGPLVLDPEEGLRLAAGKADLAADMLAM 834
P_syringae_pv._syringae_B728      SERQLAQVVLKWTGLALRNPAPERQNEALEVHVGPLVLDHEEGLRLAAGKADLAADMLAM 834
P_fuscovaginae_SE-1               SERQLAQVVLKWTGLALRNQSQERGGDAANNASELLVLDPEEGLRLAAGKADLAADMLAM 834
P_simiae_WCS417                   SERQLAQVVLKWTGLALRNQGPERVADGLGPGVQLPVLDHEEGLRLAAGKADLAADMLAM 834
P_protegens_Pf-5                  SERQLAQVVLKWTGLALRNQGPERASERPELGLELQVLDQDEGLRLAAGKADLAADMLAM 834
P_sp._UW4                         SERQLAQVVLKWSGLALRNHSPERSGDGHGGAHDLLVLDHEEGLRLAAGKADLAADMLAM 834
P_brassicacearum_DF41             SERQLAQVVLKWTGLALRNQSPERSGDAQGGS-ELLVLDHEEGLRLAAGKADLAADMLAM 833
P_fluorescens_FW300-N2E2          SERQLAQVVLKWTGLALRNQSPERSGDAPGGS-ELLVLDHEEGLRLAAGKADLAADMLAM 833
P_fluorescens_FW300-N2C3          SERQLAQVVLKWTGLALRNQSPERNSDAQGGS-ELLVLDHEEGLRLAAGKADLAADMLAM 833
P_brassicacearum_NFM421           SERQLAQVVLKWTGLALRNQSPERSGDAQGGS-ELLVLDHEEGLRLAAGKADLAADMLAM 833
                                  .*:******:**:**:*     .             *** :********* *********

P_aeruginosa_PAO1                 LLASLAADRQAIRQARDNDDRTALLERVHRLHGATRYCGVPQLRAACQTSETLLKQNDPA 890
P_sp._WCS358                      LLASLESDREAIRAAREAHDHTTLIEQVHRLNGASRYCGVPQLRAACQRSETLLKQEHPL 895
P_syringae_pv_tomato_DC3000       LLASLDADREAIRVARASQDVQALIERIHRLHGATRYCGVPQLRTACQRAETLLKQDAPH 894
P_syringae_pv._syringae_B728      LLASLDADREAIRVARANQDVHALIERIHRLHGATRYCGVPQLRSACQRAETLLKQNAPH 894
P_fuscovaginae_SE-1               LLASLDADREAIRSAREAHDNNGLIERVHRLHGATRYCGVPQLRAACQRSETLLKQDDAN 894
P_simiae_WCS417                   LLASLEADRLAITIARDANDNNALIERVHRLHGATRYCGVPQLRAACHRAETLLKQDDAK 894
P_protegens_Pf-5                  LLASLDADREAIKAARAANDQNALIERVHRLHGATRYCGVPQLRAACQRSETLLKQEDAK 894
P_sp._UW4                         LLASLEADREAIRLAQQSDDHNALIERVHRLHGATRYCGVPQLRAACQRSETLLKQQDPK 894
P_brassicacearum_DF41             LLASLEADREAIRQASEANDHNALIERVHRLHGATRYCGVPQLRAACQRSETLLKQQDPK 893
P_fluorescens_FW300-N2E2          LLASLEADREAIRQAIEANDHNALIERVHRLHGATRYCGVPQLRAACQRSETLLKQQDPK 893
P_fluorescens_FW300-N2C3          LLASLEADREAIRQACEANDHNALIERVHRLHGATRYCGVPQLRAACQRSETLLKQQDPK 893
P_brassicacearum_NFM421           LLASLEADREAIRQASEANDHNALIERVHRLHGATRYCGVPQLRAACQRSETLLKQQDPK 893
                                  ***** :** **  *   .*   *:*::***:**:*********:**: :******:

P_aeruginosa_PAO1                 AAAALDELDKAIEALADTASATTHLSSTSLDSSEL        925
P_sp._WCS358                      AQQALDELDSAINRLACQAHLNA------------        918
P_syringae_pv_tomato_DC3000       TGDALDDLDKAIVRLETEARVTA------------        917
P_syringae_pv._syringae_B728      TEEALNDLDKAIIRLEAEARVMA------------        917
P_fuscovaginae_SE-1               AHQALDELDMAIARLAIEARVSA------------        917
P_simiae_WCS417                   AQHALDELDMAIARLASEARVNA------------        917
P_protegens_Pf-5                  AFAALDELDHAIGRLAAEARTNA------------        917
P_sp._UW4                         ASVALDELDRAINRLAAHARMNA------------        917
P_brassicacearum_DF41             AAAALEDLERAINRLASEARISA------------        916
P_fluorescens_FW300-N2E2          AADALEELERAINRLASEARISA------------        916
P_fluorescens_FW300-N2C3          AAAALEDLERAINRLASEARISA------------        916
P_brassicacearum_NFM421           ATAALEELERAINRLASEARISA------------        916
                                  :  **::*: **  *   *   :
```

B.

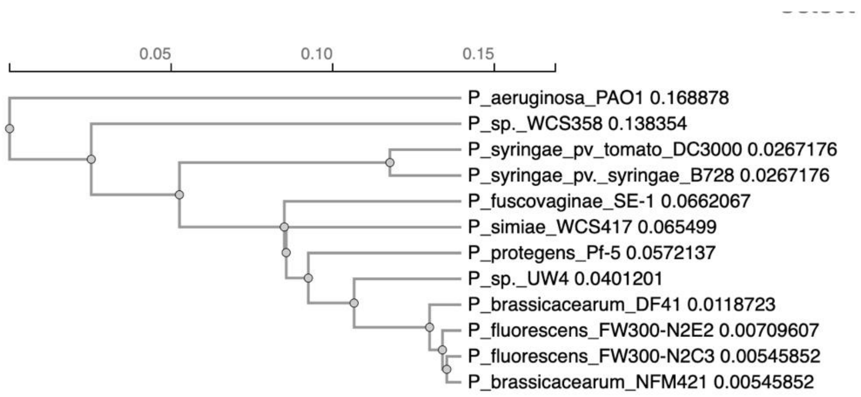

Figure S2. Alignment of GacS from strains across the genus *Pseudomonas* shows a high degree of conservation of GacS sequences. A. Clustal omega alignment of predicted GacS amino acid sequences. B. Neighbor joining tree showing relatedness of GacS sequences.

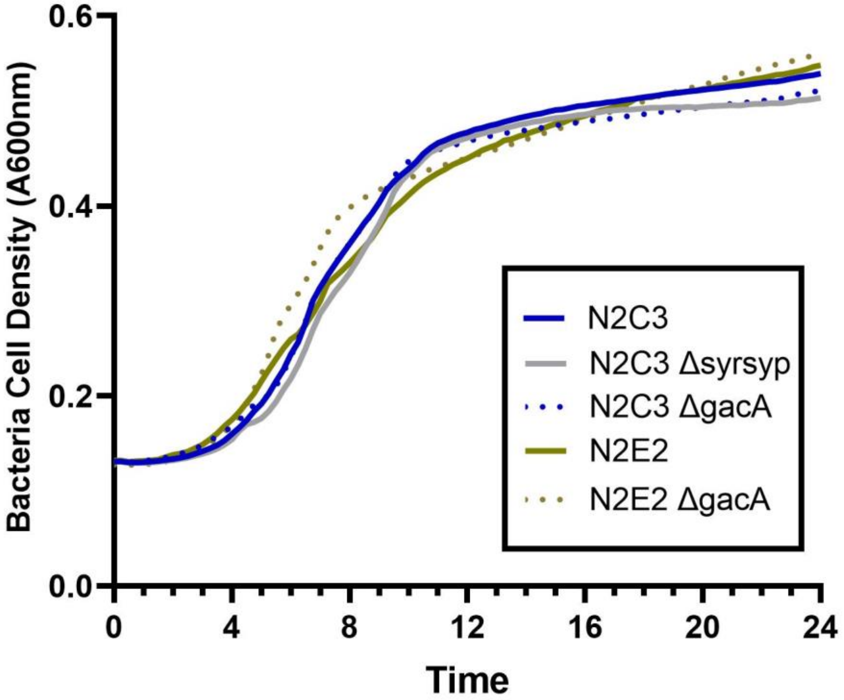

Figure S3: GacA growth curve in minimal media. A) 24-hr growth curves of each strain were performed in 1/2 MS + 20mM Succinate media with no significant differences in growth over time via repeated measures of ANOVA ($p < 0.05$; $n = 9$).
